# 

**Published:** 1894-03

**Authors:** 


### CONTENTS:

### SELECTIONS.

Article I.—Prosthetic Dentistry. By Dr. J. C. Brewer. - - 481
" II.—"Onycophagie." By Dr. Berillon. - - - 485
" III.—Teeth Below Medium in Size.—Their Treatment. By Wm. W. Belcher, D. D. S. - - - - - 492
" IV.—The World's Columbian Dental Congress. By Geo. Cunningham. - - - - - - - 499
" V.—The Relief of Pain from Diseases of the Dental Pulp and Peridental Membrane. By A. W. Harlan, M. D., D. D. S. - - - - - - - 509
" VI.—Cement Filling. By Dr. R. B. Tuller. - - 511
" VII.—Filling Roots with Chloro-Percha. By Dr. R. I. Blakeman. - - - - - - - 513
" VIII.—The Teeth of Ancient Hawaiians. By Dr. J. M. Whitney. - - - - - - - - 514
" IX.—Aluminum Alloys. By N. K. Garhart, - - 517

### EDITORIAL, Etc.

Dental Education of the Public. - - - - - - 520
The Discovery of Modern Anæsthesia. - - - - - 522

### MONTHLY SUMMARY.

Chloroform Narcosis. - - - - - - - 523

Dentists as Readers. - - - - - - - 524

The New Mineral Carborundum. - - - - - - 525

Why do Vulcanite Upper Plates Crack? - - - - - 526

Germanium. - - - - - - - - - 526

Dr. A. W. Harlan Retires. - - - - - - 526

Ancient Dentistry. - - - - - - - - 527

Dentists in Politics. - - - - - - - - 528

